# Prognostic value of androgen receptor in triple negative breast cancer: A meta-analysis

**DOI:** 10.18632/oncotarget.10208

**Published:** 2016-06-21

**Authors:** Changjun Wang, Bo Pan, Hanjiang Zhu, Yidong Zhou, Feng Mao, Yan Lin, Qianqian Xu, Qiang Sun

**Affiliations:** ^1^ Department of Breast Surgery, Peking Union Medical College Hospital, Beijing, China; ^2^ Department of Dermatology, University of California, San Francisco, CA, USA

**Keywords:** androgen receptor, breast cancer, triple negative breast cancer, prognostic value, meta-analysis

## Abstract

**Background:**

Androgen receptor (AR) is a promising therapeutic target for breast cancer. However, its prognostic value remains controversial in triple negative breast cancer (TNBC). Here we present a meta-analysis to investigate the correlation between AR expression and TNBC prognosis.

**Results:**

Thirteen relevant studies with 2826 TNBC patients were included. AR positive rate was 24.4%. AR+ patients tended to have lower tumor grade (*p*< 0.001), but more lymph node metastases (*p* < 0.01). AR positivity was associated with prolonged disease free survival (HR 0.809, 95% CI = 0.659-0.995, *p* < 0.05), but had no significant impact on overall survival (HR 1.270, 95% CI=0.904-1.782, *p* = 0.168). No difference in survival existed between subgroups using different AR or estrogen receptor cutoff values.

**Materials and methods:**

Literature search was performed in Pubmed, Embase and Cochrane Central Register of Controlled Trials databases to identify relevant articles on AR and TNBC prognosis. Fixed- and random-effect meta-analyses were conducted based on the heterogeneity of included studies. Heterogeneity and impacts of covariates were further evaluated by subgroup analyses and meta-regression.

**Conclusion:**

AR positivity is associated with lower risk of disease recurrence in TNBC. Further clinical studies are warranted to clarify its prognostic role on TNBC recurrence and survival.

## INTRODUCTION

Triple-negative breast cancer (TNBC) comprises 10-17% of breast cancer [1]. Since TNBC was insensitive to endocrine or target therapy, it was considered to have the poorest prognosis among different molecular subtypes [2, 3]. Numerous studies have been carried out on sub-classifying TNBC to find valuable prognostic and therapeutic markers. Lehmann *et al*. demonstrated that TNBC is highly heterogeneous and consists of various subgroups, such as basal-like, immune-modulatory, mesenchymal, mesenchymal stem–like, and luminal androgen receptor (LAR) subtypes [4], among which the LAR subgroup characterized with high AR expression and poor prognosis [5].

AR is expressed in 60-70% of breast cancer and approximate 0-53% of TNBC [6]. As a novel target for endocrine therapy, its therapeutic effect largely depends on estrogen receptor (ER) status. For ER+/AR+ breast cancer cell lines, AR activation had anti-proliferative effect [7–9]; in contrast, it induced pro-proliferative effect for ER-/AR+ cell lines. [4, 7, 8, 10]. Therefore, the antagonism of AR against ER may provide a new perspective for endocrine therapy.

Several clinical studies were conducted to evaluate AR prognostic value in breast cancer. It has been validated that AR positivity is associated with prolonged survival in ER+ breast cancer.[11–13]. However, the correlation between AR expression and prognosis remains elusive in TNBC. Study by Tang *et al*. suggested that absence of AR expression in TNBC served as a high-risk factor for both disease recurrence and death [14]. Similarly, several studies of (neo-) adjuvant chemotherapy reported AR positivity as an independent predictor for pathological complete response [15] and improved TNBC survival [16]. On the contrary, another cohort study revealed that AR+ TNBC had worse survival [17].

Hence, we conducted a meta-analysis including 13 studies to evaluate the prognostic value of AR in TNBC. This is the first meta-analysis to investigate the impact of AR expression on TNBC survival.

## RESULTS

**Figure 1:**
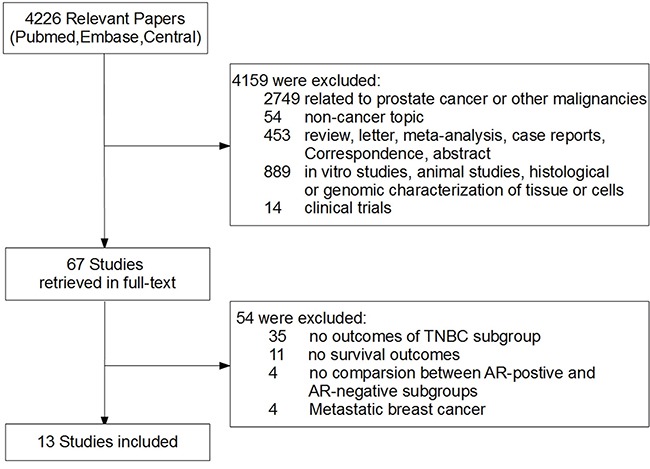
Flowchart of articles reviewed and included in meta-analysis

Among 4226 potentially relevant citations, 67 full-text articles were retrieved for detailed evaluation. Ultimately, 13 studies with 2826 patients were included in the meta-analysis (Figure 1) [13–25]. Both disease-free survival (DFS) and overall survival (OS) were available for 11 studies, while the other two studies had only DFS [22] or OS [23] reported. Supplementary Table S1 showed the quality assessment of included studies.

Table 1 summarizes the main characteristics of included studies. One study (7.7%) included only post-menopausal women, the other 12 studies (92.3 %) included both pre- and post-menopausal women. ER and progesterone receptor (PR) cutoff values were reported in seven studies (53.8%): one study (7.7%) used 0%, two studies (15.4%) used 1%, and four studies (30.8%) used 10%. Twelve studies (92.3%) used immunohistochemical (IHC) staining for AR assessment, the other one used Reverse-phase protein lysate microarray. The cutoff value for AR positivity was reported in 10 (76.9%) of 12 studies: one study (7.7%) used 0%, three studies (23.1%) used 1%, one study (7.7%) used 5%, and four studies (30.8%) used 10%.

**Table 1:** Characteristics of studies included in meta-analysis

Study	Country	N	Patients	Follow-up (m)	menopausal status	Cutoff for ER/PR(−)	AR Antibody	Assessment of AR(+)		AR(+) (%)
								Methods	Cutoff value	
**Choi 2015 [17]**	South Korea	492	Whole cohort	1-202	Pre-/Post-	IHC (<1%)	ER179(2) (Epitomics)	IHC	≥1%	87(17.7)
**Doberstein 2014 [25]**	Austria	52	Whole cohort	NR	Pre-/Post-	NR	SP107 (Ventana)	IHC	≥10%	21(40.4)
**Gonzalez-Angulo 2009 [24]**	United States	97	Subgroup	9.6-110.4	Pre-/Post-	IHC (<10%)/RPPA	AR antibody (Epitomics)	RPPA	Log mean centered value≥0.0852	16(16.5)
**He 2012 [23]**	China	287	Whole cohort	8-182	Pre-/Post-	NR	AR antibody (DAKO)	IHC	≥5%	74(25.8)
**Hu 2011 [22]**	United States	211	Subgroup	Median 168	Post-	NR	AR441 (DAKO)	IHC	≥1%	NR
**Loibl 2011 [15]**	German	111	Subgroup	1.5-96.7	Pre-/Post-	IHC (<10%)	F39.4.1 (BioGenex)	IHC	Remmele Score 3	24(21.6)
**Luo 2010 [16]**	China	137	Whole cohort	NR	Pre-/Post-	NR	NR	IHC	IHC score^a^	38(27.7)
**McGhan 2014 [21]**	United States	94	Whole cohort	0-118.8	Pre-/Post-	NR	AR441 (DAKO)	IHC	≥10%	22(23.0)
**Park 2011 [13]**	South Korea	156	Subgroup	Mean 72.7	Pre-/Post-	IHC (<10%)	AR441 (Thermo scientific)	IHC	≥10%	21(13.5)
**Pistelli 2014 [20]**	Italy	81	Whole cohort	2.5-95	Pre-/Post-	IHC (<10%)	F39.4.1 (BioGenex)	IHC	≥10%	15(18.8)
**Rakha 2007 [19]**	United kingdom	282	Subgroup	DFS 1-206 OS 1-206	Pre-/Post-	IHC (0%)	F39.4.1 (BioGenex)	IHC	≥0%	36(12.8)
**Tang 2012 [14]**	China	127	Whole cohort	DFS 10-52 OS 10-52	Pre-/Post-	NR	AR441 (DAKO)	IHC	≥10%	16(12.6)
**Thike 2014 [18]**	Singapore	699	Whole cohort	1-213	Pre-/Post-	IHC (<1%)	AR27 NCL-AR318 (Novocastra)	IHC	≥1%	267(38.0)

AR: Androgen receptor; ASCO: American Society of Clinical Oncology; DFS: Disease free survival; ER: Estrogen receptor; FISH: Fluorescence *in situ* hybridization; IHC: Immunohistochemical staining; NR: Not reported; OS: overall survival; PR: Progesterone receptor; RPPA: Reverse-phase protein lysate microarray;

a Score according to the percentage of positive cells and staining intensity [16].

Table 2 showed the correlation between AR status and clinico-pathological parameters. Twelve studies (92.3%) reported AR+ percentage. Among these twelve studies, AR expressed in 637 (24.4%) of 2615 patients. AR expression was higher in post-menopausal women (post- vs. pre-menopausal 26.9% vs 13.4%, *p* < 0.001), grade 1-2 tumor (Grade 1-2 vs. 3, 40.8% vs. 23.0%, *p* < 0.001) and patients with axillary LN metastases (LN+ vs. LN-, 28.8% vs. 22.6%, *p* < 0.01). AR expression had no significant correlation with T stage, ductal or non-ductal cancer, lymphatic vascular invasion, surgical treatment, and adjuvant chemotherapy.

**Table 2:** Correlation between androgen receptor expression and clinicopathological parameters in triple negative breast cancer patients

		AR- (%)	AR+ (%)	OR (CI)	*p* value
**Menopausal status**				0.423 (0.247-0.709)	<0.001
	Pre-	174 (86.6)	27 (13.4)		
	Post-	174 (73.1)	64 (26.9)		
**T stage**				0.846 (0.667-1.070)	0.153
	T1	438 (75.0)	146 (25.0)		
	T2-4	774 (71.7)	305 (28.3)		
**Pathology**				0.934 (0.641-1.380)	0.716
	Ductal	973 (72.9)	362 (27.1)		
	Non Ductal	113 (71.5)	45 (28.5)		
**Histological grade**				2.317 (1.806-2.969)	<0.001
	Grade 1-2	223 (59.2)	154 (40.8)		
	Grade 3	1047 (77.0)	312 (23.0)		
**LVI**				1.082 (0.841-1.394)	0.530
	No	735 (71.1)	299 (28.9)		
	Yes	335 (72.7)	126 (27.3)		
**Lymph-node metastasis**				0.723 (0.578-0.904)	<0.01
	No	558 (77.4)	163 (22.6)		
	Yes	790 (71.2)	319 (28.8)		
**Surgical treatment**				1.567 (0.919-2.748)	0.085
	Mastectomy	249 (73.9)	88 (26.1)		
	Lumpectomy	102 (81.6)	23 (18.4)		
**Chemotherapy**				1.170 (0.619-2.137)	0.584
	No	45 (72.6)	17 (27.4)		
	Yes	771 (75.7)	248 (24.3)		

AR: Androgen receptor; CI: Confidence interval LVI: Lymphovascular invasion; OR: Odds ratio;

### Disease free survival

**Figure 2:**
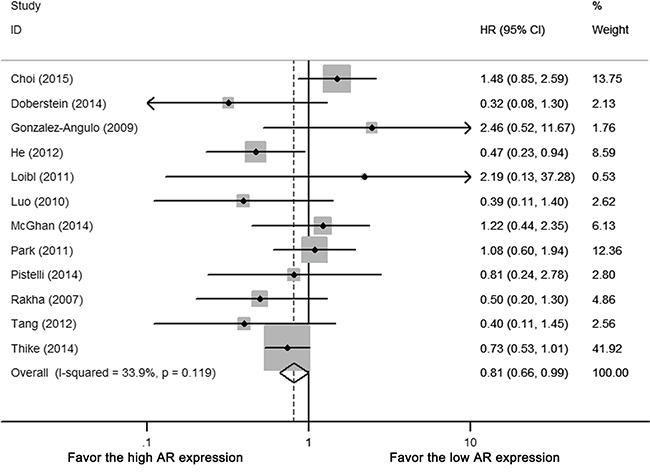
Forest plot of HR for DFS Square indicate point estimate of each study. Size of square indicates relative contribution of each study. Solid horizontal line represents 95% CI of each study. Diamond indicates pooled studies.

Twelve (92.3%) of the 13 studies reported hazard ratio (HR) of DFS, among which four studies were estimated by univariate survival analysis (log-rank test), eight studies by multivariate analysis (Cox proportional hazards model). There was no heterogeneity between included studies (Cochrane's Q *p* = 0.119, I-square = 33.9%). AR expression in TNBC was associated with improved DFS (HR 0.809, 95% CI = 0.659-0.995, *p* < 0.05) (Figure 2). For subgroup using multi-variate analysis, the pooled result of AR associated DFS remained significant (HR 0.789, 95%CI = 0.629-0.991, *p* < 0.05).

**Figure 3:**
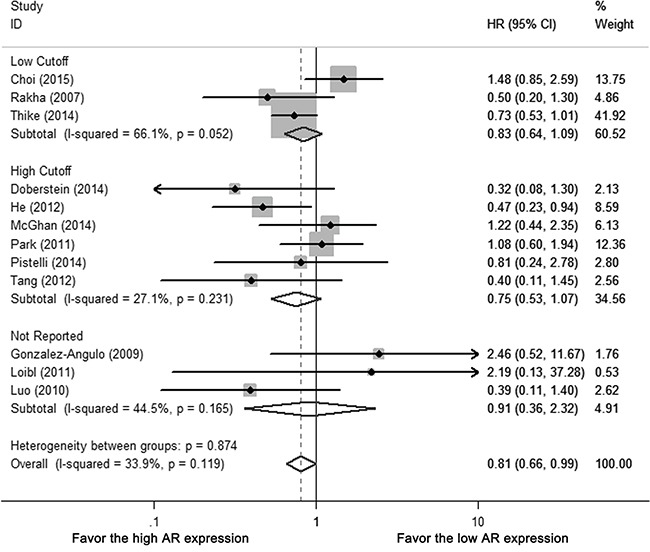
Subgroup analysis of DFS according to different AR cutoffs: low cutoffs (0 or 1%) vs. high cutoffs (10%) Size of square indicates relative contribution of each study. Solid horizontal line represents 95% CI of each study. Diamond indicates pooled HR value.

For different AR cutoffs, neither low cutoff (0 or 1%), nor high cutoff (5 or 10%) subgroups revealed AR-related benefit for DFS (HR 0.861, 95% CI = 0.494-1.503, *p* = 0.181; HR 0.754, 95% CI=0.531-1.072 *p* = 0.115, respectively) (Figure 3, Supplementary Table S2). There was no statistical difference between different AR cutoffs (*p* = 0.654), study population (whole-cohort with TNBC vs. TNBC subgroup [*p* = 0.693]), ethnicity (*p* = 0.168) or ER cutoffs (0 or 1% vs. 10%, *p* = 0.270) (Supplementary Table S2).

### Overall survival

**Figure 4:**
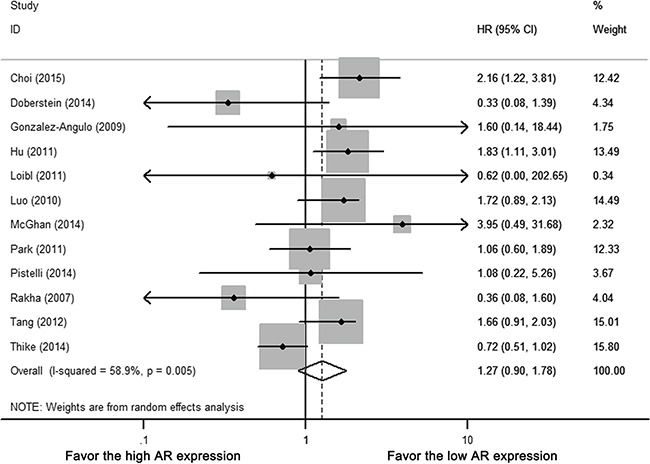
Forest plot of HR for OS Square indicate point estimate of each study. Size of square indicates relative contribution of each study. Solid horizontal line represents 95% CI of each study. Diamond indicates pooled HR value.

Among 12 studies with OS data, five studies estimated HR by univariate survival analysis, seven studies by multivariate analysis. Heterogeneity existed among included studies (Cochrane's Q *p* < 0.01, I-square = 58.9%). By random-effect meta-analysis, AR positivity was not associated with any improvement in OS (HR 1.270, 95% CI = 0.904-1.782, *p* = 0.168) (Figure 4).

**Figure 5:**
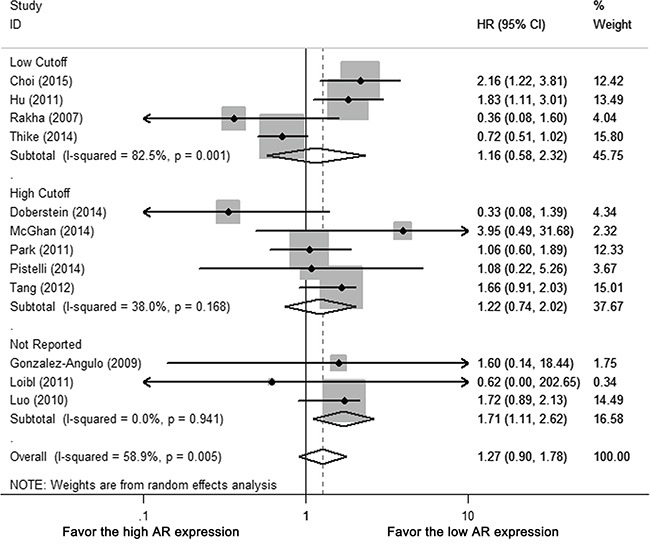
Subgroup analysis of OS according to different AR cutoffs: low cutoffs (0 or 1%) vs. high cutoffs (5 or 10%) Size of square indicates relative contribution of each study. Solid horizontal line represents 95% CI of each study. Diamond indicates pooled HR value.

For different AR cutoffs, neither low cutoff (0 or 1%) nor high cutoff (5 or 10%) subgroup revealed AR-related benefit for OS (HR 1.159, 95% CI = 0.578-2.324 *p* = 0.678; HR 1.350, 95% CI = 0.988-1.843 *p* = 0.059, respectively) (Figure 5, Supplementary Table S3). There was no statistical significance between different AR cutoffs (*p* = 0.350), study population (whole cohort vs. subgroup of cohort, *p* = 0.791), ethnicity (*p* = 0.054), ER cutoffs (0 or 1% vs. 10%, *p* = 0.654) or statistical analysis (univariate vs. multivariate, *p* = 0.165) (Supplementary Table S3). Removal of one study with post-menopausal women only [22] had no significant impact on heterogeneity of meta-analysis or pooled result of OS (I-square 59.1%, HR 1.195, 95% CI = 0.821-1.740) (Supplementary Table S3).

### Meta-regression

Meta-regression was performed on outcome data. None of the covariates showed statistically significant effects on DFS or OS (Supplementary Table S4).

### Publication bias

**Figure 6:**
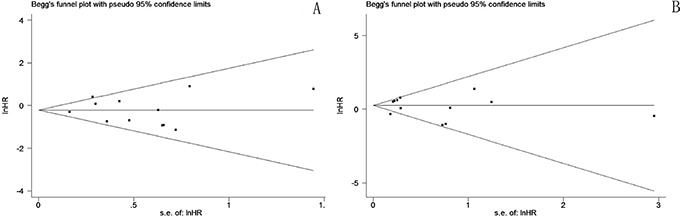
Funnel plot for potential publication bias of DFS A. and OS B

Funnel plots (Figure 6) for DFS and OS analyses were fairly symmetrical, and Begg's test revealed no significant publication bias (DFS *p* = 0.537, OS *p* = 0.945).

## DISCUSSION

As a novel therapeutic target, AR was widely investigated in many pre-clinical and clinical studies for TNBC [17, 19, 26]. However, no consensus for its correlation with TNBC prognosis has been reached to date. Hence, we conducted this meta-analysis including 13 studies and 2826 patients to evaluate AR prognostic value in TNBC. Our study revealed that AR+ TNBC was associated with lower risk of disease recurrence. To the best of our knowledge, this is the first meta-analysis on the correlation of AR expression with TNBC prognosis.

Meta-analyses were conducted to study impacts of AR on DFS and OS for all breast cancer subtypes and drew contradictory results for OS [12] [27]. Study by Qu *et al*. found that AR+ was beneficial to DFS in TNBC subgroup (HR 0.40, 95% CI 0.23-0.69), but not for OS (HR 0.90, 95% CI 0.61-1.32) [12]. Kim *et al*. suggested that AR expression benefited both DFS and OS (OR, 0.44, 95% CI, 0.26–0.75; OR, 0.26, 95% CI, 0.12–0.55, respectively) [27]. Since a limited number of studies were included in the two meta-analyses (total four retrospective studies) [14, 16, 23, 24], we conducted this meta-analysis with 13 studies and 2826 patients to provide a more reliable result. In agreement with the study by Qu *et al*., AR positivity showed no effect on OS, but lowered recurrence risk in TNBC.

Several studies revealed the antagonizing effect of AR against ER signaling depended on the relative levels of these two steroids receptors. They competed for co-regulatory molecules, resulting in opposite effects on cancer cell proliferation [28]. Without functional ER, AR may be the primary driver of downstream signaling and facilitate cancer progression [29, 30]. It implied that different AR and ER cutoffs could be potential confounding factors and sources of heterogeneity. Hence, we conducted subgroup analysis on AR/ER cutoff values, and proved they had no significant influence on final results. This was consistent with the meta-analysis by Vera-Badillo *et al*. which demonstrated AR expression incurred better prognosis irrespective of ER co-expression [11]. With ER and AR expression less than 10%, the mutual influence of ER versus AR could be negligible, therefore a suitable model to investigate AR without potential ER influence could be TNBC with ER < 10%.

Subgroup analyses showed remarkable decrease in the heterogeneity of OS in subgroups of whole-cohort TNBC patients and high AR/ER cutoffs (Supplementary Table S3), suggesting “study population” and “AR/ER cutoff values” as sources of heterogeneity. In agreement with overall result, all subgroups with different AR/ER cutoffs revealed that AR had no significant impact on TNBC OS, which provided additional evidence to AR prognostic value on OS. Moreover, the pooled results were further strengthened by data from subgroup using multi-variate analysis, since it provided more reliable data than uni-variate analysis [31].

Our studies had several limitations. First, it based on population data other than individual patient data, and restrained our ability to conduct analyses for LN metastases and other covariates. Second, all the studies were retrospective. It could potentially increase certain bias, such as selection bias. Third, we were unable to identify correlation between AR and molecular intrinsic subtypes of TNBC, especially for LAR subtype which may be helpful to clarify AR prognostic value.

In conclusion, although variability and heterogeneity existed among included studies, AR expression was associated with lower risk of disease recurrence. Since AR had a relatively high prevalence in TNBC, evaluating AR status could provide additional information on prognosis and AR-targeting therapy could be another option for chemotherapy resistant TNBC in both neoadjuvant and metastatic settings. Future clinical trials based on different molecular subtypes of TNBC are warranted to further clarify its prognostic value and efficacy of AR-targeting therapy.

## MATERIALS AND METHODS

### Data sources and search strategy

We searched the following databases for relevant studies: PubMed (from 1946 to July, 2015), Embase (host: Ovid, from 1947 to July, 2015) and Cochrane Central Register of Controlled Trials (CENTRAL, from 2000 to July, 2015). The following medical subject headings, Embase Emtree terms and keywords were used: ‘Breast Neoplasms’, ‘Triple Negative Breast Neoplasms’, ‘Breast Cancer’, ‘Triple Negative Breast Cancer’, and ‘Androgen Receptor’. There was no limitation on languages or regions of publications. Reference lists of all the relevant articles were manually screened to ensure the sensitivity of the literature search.

### Selection criteria and quality assessment

To be eligible, studies had to meet the following inclusion criteria: TNBC patients, or studies that provided data on TNBC as a subgroup; assessment of AR expression in primary breast cancer tissue; available data of OS or DFS on patient subgroups with different AR level. Exclusion criteria included: metastatic disease; review, meta-analysis, editorial, letter or conference abstract. Two independent reviewers (C.J. Wang and B. Pan) evaluated eligibility of studies according to the above criteria. Full text of the potentially relevant studies were obtained and reviewed for inclusion by the same two reviewers. Disagreement was resolved by consensus (C.J. Wang, B. Pan, Q. Sun). Inter-reviewer agreement was assessed by Kappa Statistics according to Higgins *et al*. [32]

The quality of the included studies was assessed according to the STROBE checklist for Cohort Studies [33]. Each item in the STROBE Checklist was scored using an ordinal scale (1-5, with 1=Worst, 5=Best) by two independent reviewers (C.J. Wang and B. Pan). The final quality scores were the average of scores generated by each reviewer and expressed as percentages, ranging 0–100%, where higher values indicated a better methodological quality.

### Data extraction

Data were collected using a predesigned data extraction form by two reviewers (C.J Wang and B. Pan). Characteristics of included studies were extracted for stratified analyses and meta-regression. Survival data (HR with CI and *p*-value) were extracted from tables or text of included studies, or estimated from Kaplan-Meier curves where applicable [34].

### Statistical analysis

Statistical variables such as HR and corresponding CI were directly taken and used for the meta-analysis. Other data were presented as means and proportions, differences between groups were tested with Pearson Chi-square test. The HR of OS and DFS was chosen as the primary analytical endpoint. Fixed effects or random effects models were used based on whether significant heterogeneity existed between studies.

Publication bias was assessed by Begg's test and symmetry of funnel plot. Statistical heterogeneity was assessed by Cochrane's Q and I-square statistics. Cochrane's Q test with *p* < 0.05 or I-square >50% indicated significant heterogeneity among included studies. To investigate potential heterogeneity across studies, subgroup analyses were performed according to methods described by Deeks *et al*. [31]. Meta-regression was conducted for all potential confounding factors. All the statistical tests were two-sided, and statistical significance was defined as *p* < 0.05. Statistical analyses were conducted by STATA version 12.0 (Stata Corporation, College Station, TX, USA).

## SUPPLEMENTARY TABLES


