# Supplementary material for: Prognostic value of androgen receptor in triple negative breast cancer: A meta-analysis
Source: Oncotarget. 2016 Jun 21;7(29):46482–91. doi: 10.18632/oncotarget.10208 (PMC5216811; doi:10.18632/oncotarget.10208)
Supplement: Supplementary file 1 [file oncotarget-07-46482-s001.pdf]

Prognostic value of androgen receptor in triple negative breast cancer: A meta-analysis

SUPPLEMENTARY TABLES

Supplementary Table S1: Quality assessment of included studies

Author	Year	C.J. Wang (reviewer1)	B. Pan (reviewer2)	Average
Choi *et al.*	2015	97.27	96.36	96.82
Doberstein *et al.*	2014	91.82	94.55	93.19
Gonzalez-Angulo *et al.*	2009	94.55	93.64	94.10
He *et al.*	2012	92.73	91.82	92.28
Hu *et al.*	2011	93.64	92.73	93.19
Loibl *et al.*	2011	100	99.09	99.55
Luo *et al.*	2010	96.36	94.55	95.46
McGhan *et al.*	2014	94.55	95.45	95.00
Park *et al.*	2011	98.18	97.27	97.73
Pistelli *et al.*	2014	95.45	95.45	95.45
Rakha *et al.*	2007	97.27	97.27	97.27
Tang *et al.*	2012	98.18	96.36	97.27
Thike *et al.*	2014	98.18	97.27	97.73

Quality score was generated according to the items in STROBE Checklist. Each item in the STROBE Checklist was scored using an ordinal scale (1-5, with 1=Worst, 5=Best) by two independent reviewers (C.J. Wang and B. Pan). The final quality scores (QS) were the average of scores generated by each reviewer and expressed as percentages, ranging 0–100%.

Supplementary Table S2: Subgroup analysis of DFS

**Overall**		**I-square (33.9%)**	**HR (0.809)**	**Lower CI (0.659)**	**Upper CI (0.995)**	***p* value (<0.05)**	**Model (Fixed)**
**Participants**	**Between groups**					0.693	
	Subgroup	43.0%	0.792	0.627	1.000	0.050	Fixed
	Whole cohort	32.8%	0.876	0.562	1.365	0.558	Fixed
**Ethnicity**	**Between groups**					0.168	
	Asian	52.6%	0.803	0.639	1.009	0.059	Fixed
	European	0.0%	0.559	0.294	1.062	0.076	Fixed
	American	0.0%	1.426	0.684	2.971	0.344	Fixed
**ER cutoff**	**Between groups**					0.270	
	Low (0 or 1%)	66.1%	0.861	0.494	1.503	0.181	Random
	High (10%)	0.0%	1.144	0.698	1.874	0.593	Fixed
**AR Cutoff**	**Between groups**					0.654	
	Low (0 or 1 %)	66.1%	0.861	0.494	1.503	0.181	Random
	High (5 or 10%)	27.1%	0.754	0.531	1.072	0.115	Fixed
**Statistical Analyses**	**Between groups**					0.605	
	Multivariate	48.1%	0.789	0.629	0.991	< 0.05	Fixed
	Univariate	0.0%	0.910	0.558	1.483	0.704	Fixed

AR: Androgen receptor; HR: Hazard ratio; CI: Confidence interval;

Supplementary Table S3: Subgroup and sensitivity analysis of OS

Overall		I-square (58.9%)	HR (1.270)	Upper CI (0.904)	Lower CI (1.782)	*p* value (0.168)	Model (Random)
**Participants**	**Between groups**					0.791	
	Whole Cohort	41.7%	1.227	0.865	1.741	0.250	Fixed
	Subgroup	72.4%	1.451	0.914	2.305	0.115	Random
**Ethnicity**	**Between groups**					0.054	
	Asian	74.0%	1.409	0.975	2.035	0.068	Random
	European	0.0%	0.487	0.207	1.148	0.100	Fixed
	American	0.0%	2.698	0.553	13.164	0.220	Fixed
**Design**	**Between groups**					NA	
	Retrospective	59.1%	1.195	0.821	1.740	0.351	Random
	Prospective [a]	NA	NA	NA	NA	NA	
**Menopausal status**	**Between groups**					NA	
	Pre-/Post-	59.1%	1.195	0.821	1.740	0.351	Random
	Post- only[a]	NA	NA	NA	NA	NA	
**ER cutoff**	**Between groups**					0.654	
	Low (0 or 1%)	83.4%	0.946	0.374	2.393	0.907	Random
	High (10%)	0.0%	1.077	0.637	1.821	0.781	Fixed
**AR cutoff**	**Between groups**					0.350	
	Low (0 or 1 %)	82.5%	1.159	0.578	2.324	0.678	Random
	High (5 or 10%)	38.0%	1.350	0.988	1.843	0.059	Fixed
**Statistical Analyses**	**Between groups**					0.165	
	Multivariate	69.0%	1.420	0.947	2.128	0.090	Random
	Univariate	0.0%	0.916	0.554	1.515	0.733	Fixed

AR: Androgen receptor; CI: Confidence interval; ER: Estrogen receptor; HR: Hazard ratio; NA: not applicable;
[a] Only one study included in the one or more subgroups, subgroup analysis was unable to perform.

Supplementary Table S4: Meta regression result of DFS and OS (listed is the *P* value for each covariate)

	DFS (*p* value)	OS (*p* value)
Ethinicity	0.411	0.763
ER Cutoff	0.118	0.983
Study Design	Not available [a]	0.744
Assessment method of AR	0.928	0.755
AR Cutoffs	0.788	0.735
Statistical Analyses (Univairate/Multivariate)	0.377	0.497

AR: Androgen receptor; DFS: Disease free survival; OS: Overall survival.

[a] “Study Design” in DFS and “Menopausal Status” were dropped due to colinearity.
